# Modeling of Task Planning for Multirobot System Using Reputation Mechanism

**DOI:** 10.1155/2014/818701

**Published:** 2014-02-04

**Authors:** Zhiguo Shi, Jun Tu, Yuankai Li, Junming Wei

**Affiliations:** ^1^School of Computer and Communication Engineering, University of Science and Technology, Beijing 100083, China; ^2^Department of Aerospace Engineering, Ryerson University, 350 Victoria Street, Toronto, ON, Canada M5B 2K3; ^3^School of Aeronautics and Astronautics, University of Electronic Science and Technology of China, Chengdu 611731, China; ^4^ANU College of Engineering and Computer Science, Australian National University, Canberra, ACT 2601, Australia

## Abstract

Modeling of task planning for multirobot system is developed from two parts: task decomposition and task allocation. In the part of task decomposition, the conditions and processes of decomposition are elaborated. In the part of task allocation, the collaboration strategy, the framework of reputation mechanism, and three types of reputations are defined in detail, which include robot individual reputation, robot group reputation, and robot direct reputation. A time calibration function and a group calibration function are designed to improve the effectiveness of the proposed method and proved that they have the characteristics of time attenuation, historical experience related, and newly joined robot reward. Tasks attempt to be assigned to the robot with higher overall reputation, which can help to increase the success rate of the mandate implementation, thereby reducing the time of task recovery and redistribution. Player/Stage is used as the simulation platform, and three biped-robots are established as the experimental apparatus. The experimental results of task planning are compared with the other allocation methods. Simulation and experiment results illustrate the effectiveness of the proposed method for multi-robot collaboration system.

## 1. Introduction

The field of distributed robotics started in the late 1980s, when several researchers began investigating issues in multiple mobile robot systems. Prior to this time, researches had concentrated on either single robot systems or distributed problem-solving systems that did not involve robotic components [1]. In the early distributed robotics work, the topics of particular interest include  (1) cellular (or reconfigurable) robot systems, such as cellular robotic system [2] and cyclic swarms [3]; (2) multirobot motion planning, such as traffic control [4] and movement in formation [5]; (3) architectures for multirobot collaboration, such as ACTRESS [6]. Also, literatures on other particular topics, such as robot colonies [7] and heterogeneous multirobot systems [8], have been published.

In recent years, multirobot systems research has made great progress in many areas [9]. Issues of multirobot system [10] include biological inspirations, communication, architectures, localization/mapping/exploration, object transport and manipulation, motion coordination and formation [11, 12], reconfigurable robots, and learning.

Collaboration is an important characteristic and a major evaluation indicator for multirobot systems [6]. Multirobot systems can complete the mission through collaboration which a single robot cannot achieve. It is not the linear summation of the role of single robot but includes the individual-based interactively incremental besides the linear summation, which increases the overall performance [13].

Task planning includes two aspects [9]: task decomposition and task allocation. Presently, task planning system has been widely used in the space shuttle satellites, pilotless aircraft, and so forth. The research of multirobot systems is closely related to the distributed artificial intelligence, which consists of two main areas of research: distributed problem solving (DPS) and multiagent systems. DPS mainly focuses on how to decompose a particular problem and how to solve the problem among multiple agents, including three main aspects, which are problem decomposition, subproblem solving, and synthesis of the findings.

In the task decomposition and distribution research, there are several traditional methods: Contract Net Mechanism [14], Local/Global Planning [15], Distributed Searching, and Joint Intentions. Contract Net Protocol is an important noncentralized task decomposition and distribution model in distributed artificial intelligence.

The traditional task allocation is mainly based on contract net protocol (CNP) [14], which can provide the solution for the issues by consulting to avoid conflict. Parker [16] developed a behavior-based distributed multirobot collaboration structure named L-ALLICANCE and the corresponding system with the capability of parameters learning named L2-ALLICANCE [17] in MIT. The system utilizes an incentive-based task allocation mechanism for behavior and enhances the generalization and error tolerance of multirobot system. Parker also proposed a behavior-based task allocation mechanism named ASyMTRe [18].

Gerkey applied the market algorithm in multirobot system to deal with dynamic task allocation problem named MURDOCH [19]. The market mechanism in task allocation is based on consultations. The robots complete the task assigned through mutual consultation and negotiation on the basis of certain agreement in multirobot system.

However, the task allocation methods have their limitations to some extent. Behavior-based task allocation mechanism does not take into account the influence of collaboration history, while market-mechanism-based task allocation does not fully consider the impact of the time factor, leading to poor robustness.

In addition, the topics of multirobot search and rescue, cooperative localization, motion coordination, and formation control attract a lot of researchers in the fields of robot collaboration system [20]. Collaboration optimization method, including linear programming method and the Hungarian algorithm [21], can be applied to solve simple tasks and robot collaboration optimization problems. But when the number of robots and tasks increases in the system, the computational complexity will increase exponentially.

In this paper, a task planning method based on reputation mechanism is proposed. Reputation plays an important role in the collaboration among people. In many cases, task allocation is based on someone's reputation, which is gained from the evaluation of the completion of historical tasks. Reputation theory is attracting interest from industrial and academic research communities and increasingly being integrated with online services and applications, especially in network computing system.

Reputation of robots is the overall assessment and the summary of past actions observed from one robot to another through the gradual dynamic capabilities in a continuous interactive process. The assessment can be used to guide further actions of the robot. Reputation includes five attributes:  (1) the related characteristics of environmental context, (2) dynamic characteristics of changing over time, (3) time lag characteristics of forming through the continuous learning and historical experience, (4) group characteristics of being affected by the group or alliance of the robot, and (5) incentive and reward mechanism. The robot with good reputation should be rewarded in task allocation. Otherwise, it should be punished.

The rest of the paper is organized into four sections as follows: the system structure and basic concepts are presented in Section 2. The modeling of task planning using reputation mechanism is proposed in Section 3. Simulation and experimental results are shown in Section 4, and concluding remarks are in Section 5.

## 2. Basic Concepts and System Structure

Task planning consists of two parts: decomposition and allocation. Task allocation in the multirobot system is divided into two categories: direct allocation and delegation allocation. Direct allocation is to assign a task to robot that can provide collaboration directly. If the robot cannot complete the assigned task, the assigned task can be delegated to other robots. The task can be further delegated if needed.

**Figure 1:**
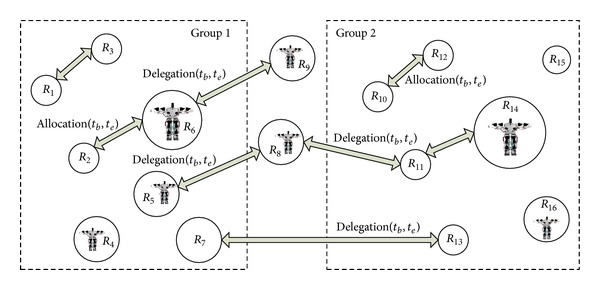
Robot collaboration system.

The periodical constraint is utilized to guarantee the time effectiveness of task allocation. (*t*_*b*_, *t*_*e*_) is a periodical constraint from *t*_*b*_ to *t*_*e*_, which means that the task allocation is valid in the period from the time *t*_*b*_ to *t*_*e*_. If a task cannot be accomplished in the arranged time, it will be withdrawn, which can improve the performance of the system. A typical collaboration system is shown in Figure 1.

In Figure 1, there are two cooperative groups. Tasks can be assigned by direct allocation or indirect delegation.

**Figure 2:**
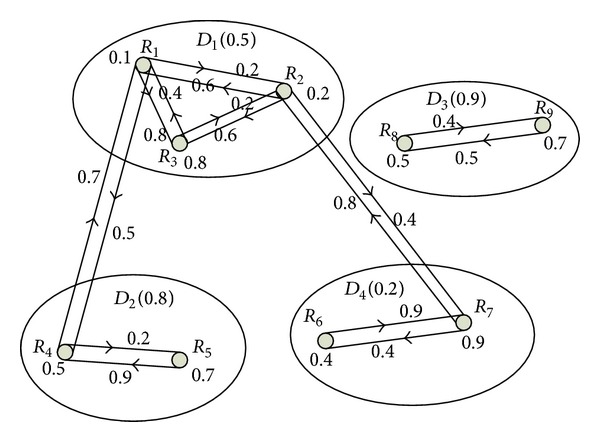
Reputation of the robot collaboration system.

In the robot reputation system, robot reputation level (RRL) is proposed to quantify the reputation of each robot, which is based on the historical experience and the overall evaluation of the system. “0” and “1” are used to quantize the reputation value. “0” represents the lowest reputation and “1” represents the highest reputation. The symbol of robots group is indicated as *D*. The value on the line from robot *R*_1_ to robot *R*_2_ is the direct reputation from *R*_1_ to *R*_2_, and the individual reputation value of the robot *R*_1_ is the value on the robot, shown in Figure 2.

In Figure 2, the reputation of the group is the value beside the name of the group; for example, the reputation of group *D*_1_ is 0.5. The reputation of robots in the collaboration system consists of three parts: (1) the individual reputation; (2) the group's reputation where the robot belongs to; (3) the reputation between the two robots intending to cooperate.

## 3. Task Planning Using Reputation Mechanism

In this section, task planning is divided into two parts: task decomposition and task allocation. Firstly, the condition and process of the task decomposition are presented. Secondly, task allocation using reputation mechanism is defined and presented in detail.

### 3.1. Task Decomposition

A task can be decomposed if it meets certain conditions. Four task decomposition conditions are given in [22]. If the task can be decomposed and is assignable, the formal definition of automata is as follows.

An automata is a tuple *A* = (*Q*, *q*_0_, *E*, *δ*, *F*), consisting of a set of states *Q*; the initial state *q*_0_; a set of events *E* that causes transitions between the states; a transition relation *δ*; *F* is the acceptable states set. *s* = *a*_0_ *a*_1_ ⋯ *a*_*n*−1_ ∈ E* is a finite alphabet and a sequence *q*_0_, *q*_1_,…, *q*_*n*_ of *n* + 1 states in *Q*, and *q*_0_ ∈ *Q*_0_, *q*_*i*+1_ ∈ *δ*(*q*_*i*_, *a*_*i*_).

The parallel composition of *A*_1_ and *A*_2_ is the automaton *A*_1_||*A*_2_ = (*Q*, *q*_0_, *E*, *δ*), defined as follows:

(1) Q=Q1×Q2;q0=(q10,q20);  E=E1∪E2;    ∀(q1,q2)∈Q,e∈Eδ((q1,q2),e)={δ1(q1,e),δ2,(q2,e)if  δ1(q1,e), δ2,(q2,e),   e∈E1∩E2(δ1(q1,e),q2)if  δ1(q1,e)!, e∈E1∖E2(q1,δ2(q2,e))if  δ2(q2,e)!, e∈E2∖E1ϕotherwise.

The natural projections of *A*_*S*_ in local event sets *E*_*i*_ is obtained as *P*_*i*_ (*A*_*S*_), merging the events-related states which belong to the event sets *E*/*E*_*i*_.

(*1) Decomposition Condition 1*

(2) ∀e1,e2∈E,  q∈Q[δ(q,e1)!∧δ(q,e2)!]⟹[∃Ei∈{E1,…,En},{e1,e2}⊆Ei]∨[δ(q,e1e2)!∧δ(q,e2e1)!],

where, *e*_1_, and *e*_2_ are events; *E* is a set of total events; local event sets *E*_*i*_; a set of states *Q*; a transition relation *δ*; If state *q* is reachable under events *e*_1_, *e*_2_ that means existing a local events *E*_*i*_ which contains events *e*_1_, *e*_2_, making the state *q* remain reachable under the event sequence *e*_1_ *e*_2_ and *e*_2_ *e*_1_.

(*2) Decomposition Condition 2*

(3) ∀e1,e2∈E, q∈Q, s∈E∗[δ(q,e1e2s)!∨δ(q,e2e1s)!]⟹[∃Ei∈{E1,…,En},{e1,e2}⊆Ei]∨[δ(q,e1e2s)!∧δ(q,e2e1s)!],

where E* is sequence of events. If the state *q* is reachable under the event sequence *e*_1_ *e*_2_ or *e*_2_ *e*_1_, it can launch a local existing events *E*_*i*_, which contains events *e*_1_, *e*_2_, making the state *q* remain reachable under the event sequence *e*_1_ *e*_2_ *s* and *e*_2_ *e*_1_ *s*.

(*3) Decomposition Condition 3*

(4) δ(q0,|i=1npi(si))!, ∀{s1,…,sn}∈L~(AS),∃si,sj∈{s1,…,sn}, si≠sj,

where $\overset{\sim }{L}(A_{S})\subseteq L(A_{S})$ and $\overset{\sim }{L}(A_{S})$ are the biggest subset of *L*(*A*_*S*_)  $\text{for}\;\text{all}\;s\in \overset{\sim }{L}(A_{S})$, $\exists s^{\prime }\in \overset{\sim }{L}(A_{S})$, ∃*E*_*i*_, *E*_*j*_ ∈ {*E*_1_,…, *E*_*n*_}, *i* ≠ *j* can launch *p*_*E*_*i*_∩*E*_*i*__(*s*) and *p*_*E*_*i*_∩*E*_*i*__(*s*′), beginning with the same event. *L*(*A*_*S*_) is the set of all sequences of events of automaton *A*_*S*_; $\overset{\sim }{L}(A_{S})$ is the set of the sequences of events of automata *A*_*S*_; the natural projections of the *i*th local event set in the corresponding sequence of events *s*_*i*_.

(*4) Decomposition Condition 4*

(5) ∀i∈{1,…,n},x·x1·x2∈Qi,x1≠x2,  e∈Ei,t∈Ei∗,δi(x,e)=x1,δi(x,e)=x2δi(x1,t)!⟺δi(x2,t)!.

Formal linear temporal logic language is used to present the whole tasks of the robot system, which is almost the same with the natural language in structure.

Several temporal operators of task decomposition are presented as follows [22]:

- next state “*ο*”: requires that a property hold in the next state of the path;
- until “∪”: used to combine two properties. The combined property holds if there is a state on the path where the second property hold, and at every preceding state on the path, the first property holds;
- eventually “⋄”: used to assert that a property will hold at some future state on the path;
- always “□”: specifies that a property holds at every state on the path. Such as: *E* = {*E*_1_, *E*_2_,…, *E*_*n*_}.

The reachability while avoiding some events: ¬  (*E*_1_∨*E*_2_) ∪ *E*_3_

 Sequence: ⋄(*E*_1_∧⋄(*E*_2_∧⋄*E*_3_))
 Coverage: ⋄*E*_1_∧⋄*E*_2_∧⋄*E*_3_
 Recurrence: □(⋄*E*_1_∧⋄*E*_2_∧⋄*E*_3_).

The equivalent task automata *A*_*S*_ can be transformed from the formula. The natural projections of *A*_*S*_ to local event sets *E*_*i*_ are obtained as *P*_*i*_ (*A*_*S*_). Linear temporal logic and automata have a close relationship, which can be directly exported from linear temporal logic formula, because every linear temporal logic formula consists of connectors and temporal operators. 

### 3.2. Reputation Mechanism

The negotiation process of the robot collaboration system is presented, which mainly is concerned about how to build partnerships between the collaborative robots according to the reputation mechanism.

**Definition 1 (robot initialization set)**

The robot initialization set *I* is identified as 〈*κ*_*I*_, *E*_*I*_, CHAP_*I*_, AC_*I*_〉, where *κ* denotes the ontology of collaboration robot, which can get the identification corresponding to *κ*; *E* ⊂ *ε* is an identification set, these identification sets are issued to *κ* in *I*. CHAP: *τ* → *P* is a partial order function, mapping a finite subset of attributes to the strategy set. The properties of CHAP are called limited properties. For the limited property *t*, CHAP[*t*] is called the verification strategy of *t*. AC: *R* → *P* is a partial order function, mapping resources to a finite strategy set.

**Definition 2 (robot collaboration strategy)**

A collaboration strategy is defined as a quintuple 〈*Q*, *M*, init_*R*_, start_*M*_, reply〉, where *Q* is the finite set of the collaboration process state, each state denoted by *q*, and, *q* ∈ *Q*; *M* is the finite set of the collaboration process messages. The massage is denoted by *m* and usually consists of subscripts. The message sequences can be denoted as *m*_1_,…, *m*_*n*_. The function init_*R*_: *I* × *k* → *Q* defines the initial state of the requester. When the initialization set and requesting robot are given, this state is init_*R*_(*I*, *K*_*R*_) = *q*, where *q* ∉ {success, failure}. start_*M*_: *I* × Res × *k* → *Q* × *M* defines that the collaboration is started by a requested robot. The function reply: *Q* × *M* → *Q* × *M* defines each action of the robot. When the robot initialization set *I*, current state *q* and the latest message *m* from the other side are given, reply(*q*, *m*) = 〈*q*′, *m*′〉.

**Definition 3 (framework of reputation mechanism)**

Giving that *D*_1_ denotes the domain where *R*_1_ belongs to, *D*_2_ denotes the domain where *R*_2_ belongs to, *t* denotes the time of collaboration and *l* denotes the specific group. The collaboration relationship from *R*_1_ to *R*_2_ is expressed as Γ(*R*_1_, *R*_2_, *t*, *c*, *l*):

(6) Γ(R1,R2,t,l,c)=α×Θ(R1,R2,t,l,c) +β×Ω(R2,t,l,c)+θ×Ψ(D1,D2,t,c),

where Θ(*R*_1_, *R*_2_, *t*, *l*, *c*) denotes the direct reputation between robot *R*_1_ and robot *R*_2_. *α* denotes the direct reputation weight coefficient, 0 ≤ *α* ≤ 1. *Ω*(*R*_2_, *t*, *l*, *c*) denotes the individual reputation value of robot *R*_2_; *β* is the reputation weight coefficient. 0 ≤ *β* ≤ 1. Ψ(*D*_1_, *D*_2_, *t*, *c*) denotes the reputation value from the robot group *D*_1_ to the group *D*_2_. *θ* denotes the group weight coefficient, 0 ≤ *θ* ≤ 1, *α* + *β* + *θ* = 1, *α*, *β*, *θ* ≥ 0.

**Definition 4 (direct reputation)**

Giving that *D*_1_ denotes the domain where *R*_1_ belongs to, *D*_2_ denotes the domain where *R*_2_ belongs to. The direct reputation from *R*_1_ to *R*_2_ is presented as

(7) Θ(R1,R2,t,l,c)=DRM(R1,R2,t,c) ×γ(t−ta−b,c)×τ(la,lb,c),

where the definition of DRM (Direct Reputation Matrix) can be seen in Definition 7. *t*_*a*−*b*_ denotes the last collaboration time between *R*_1_ and *R*_2_; *γ*(*t* − *t*_*a*−*b*_, *c*) denotes the function of time calibration; *τ*(*l*_*a*_, *l*_*b*_, *c*) denotes the function of group calibration function; and *γ*(*t* − *t*_*a*−*b*_, *c*) denotes the time calibration function.

**Definition 5 (individual reputation)**

Giving that *D*_1_ denotes the domain where *R*_1_ belongs to, *D*_2_ denotes the domain where *R*_2_ belongs to. The individual reputation of robot *R*_2_ in the collaboration system is presented as

(8) Ω(R2,t,l,c)=1n∑i=1nS(ρi+Γi(Ri,R2,t,l,c))×γ(t−tzi−b,c)×τ(lzi,lb,c),

where *S*(*ρ*_*i*_ + Γ_*i*_(*R*_*i*_, *R*_2_, *t*, *l*, *c*)) denotes the function of the reputation calibration; *ρ*_*i*_ denotes the standard deviation of reputation value at the No. *i* collaboration; *λ*(*t* − *t*_*z*_*i*_−*b*_, *c*) denotes the function of time calibration; *τ*(*l*_*z*_*i*__, *l*_*b*_, *c*) denotes the function of group calibration function; and *n* denotes the number of robots in the collaboration system who is cooperating with robot *R*_2_.

**Definition 6 (group reputation)**

Giving that *D*_1_ denotes the domain where *R*_1_ belongs to, *D*_2_ denotes the domain where *R*_2_ belongs to. Group reputation in the collaboration system is

(9) $$\begin{array}{c} \Psi \left(D_{1},D_{2},t,c\right)=\Psi \left(D_{1},D_{2},t_{a-b},c\right)\times \gamma \left(t-t_{a-b},c\right), \end{array}$$

where Ψ(*D*_1_, *D*_2_, *t*_*a*−*b*_, *c*) denotes the group reputation value of the last collaboration between the group *D*_1_ and the group *D*_2_. *γ*(*t* − *t*_*a*−*b*_, *c*) denotes the function of time calibration.

**Definition 7 (direct reputation matrix (DRM))**

If the cooperative system is composed by *n* robots, DRM will be *n*∗*n* matrix. Direct reputation value Θ(*R*_1_, *R*_2_, *t*, *l*, *c*), Θ ∈ [0,1]. For example, there are 26 robots in the collaboration system, named from *R*_*A*_ to *R*_*Z*_, the DRM of which is the following matrix:

(10) $$\begin{array}{c} \left[\begin{array}{cccc} 1 & \Theta \left(R_{A},R_{B},t,l,c\right) & \cdots & \Theta \left(R_{A},R_{Z},t,l,c\right)\\ \Theta \left(R_{B},R_{A},t,l,c\right) & 1 & \cdots & \Theta \left(R_{B},R_{Z},t,l,c\right)\\ \cdots & \cdots & \cdots & \cdots \\ \Theta \left(R_{Z},R_{A},t,l,c\right) & \Theta \left(R_{Z},R_{B},t,l,c\right) & \cdots & 1 \end{array}\right]. \end{array}$$

**Definition 8 (time calibration function)**

Giving that *D*_1_ denotes the domain where *R*_1_ belongs to, *D*_2_ denotes the domain where *R*_2_ belongs to.   *t*_*a*−*b*_ denotes the last collaboration time between robot *R*_1_ and robot *R*_2_. The time calibration function can be expressed as

(11) γ(t,c)=k0γ(ta−b,c)+k1e−k(t−ta−b)(t−ta−bTr+)S −k2e−k(t−ta−b)(t−ta−bTr−)(S−1) −k3(1−e−k(t−ta−b))γ(ta−b,c),

where *k*_0_ denotes the history experience coefficient, generally, *k*_0_ = 1. *k*_1_ denotes the reputation reward coefficient. *e* ^−*k*(*t*−*t*_*a*−*b*_)^ denotes the correction operator. *k*_2_ denotes the reputation penalty coefficient. *r* ^+^ denotes the incentive ratio in time *T*. *r* ^−^ denotes the penalty ratio in time *T*. *S* denotes validation coefficient of the collaboration, if the assigned task is successfully finished, *S* = 1; otherwise *S* = 0. *k*_3_ denotes the attenuation coefficient.

If the robot does not take any assigned task, its reputation will diminish as the time went on. The coefficients is satisfied with 0 < *k*_0_, *k*_1_, *k*_2_, *k*_3_ ≤ 1, *k* > 0.

*Characteristic 1 *(time attenuation). With no compensation, the proposition will be proved if *γ*(*t*_1_) > *γ*(*t*_2_), when *t*_1_ < *t*_2_

(12) $$\begin{array}{c} \gamma \left(t_{1},c\right)=k_{0}\gamma \left(t_{0},c\right)-k_{3}\left(1-e^{-k(t_{1}-t_{0})}\right)\gamma \left(t_{0},c\right),\\ \gamma \left(t_{2},c\right)=k_{0}\gamma \left(t_{0},c\right)-k_{3}\left(1-e^{-k(t_{2}-t_{0})}\right)\gamma \left(t_{0},c\right),\\ \gamma \left(t_{1},c\right)-\gamma \left(t_{2},c\right)=k_{3}\gamma \left(t_{0},c\right)\left(e^{-k(t_{1}-t_{0})}-e^{-k(t_{2}-t_{0})}\right), \end{array}$$

where *k*_3_ *γ*(*t*_0_) > 0 and *t*_1_ < *t*_2_, so the equation above is greater than 0; therefore, the proposition may be established. In the process of collaboration, if no compensation, the value of the function will be decreased.

*Characteristic 2* (natural attenuation curve). Generally, *k*_0_ is 1. The value of (11) is related to *k* and *k*_3_. Taking that *k* > 0,  0 < *k*_3_ ≤ 1, a different set of curves will be obtained, which are called the attenuation curves.

The curve, when *k* = 0.2, *k*_3_ = 0.5, is defined as the natural attenuation curve. Assuming that the initial value *k*_0_ *γ*(*t*_0_, *c*) = 1, *k*_3_ = 0.5 could be the case, *k* is 0.1, 0.2, 0.3, and 0.4, respectively.

*Characteristic 3* (historical experience related). Assuming that (11) do not have the characteristics of historical relevance, in the absence of natural attenuation as well as under the circumstances of no penalty and award compensation, reputation will have nothing to do with the historical experience. Then *k*_0_ *γ*(*t*_*a*−*b*_, *c*) = 0, because *γ*(*t*_*a*−*b*_, *c*) is not equal to zero, so *k*_0_ = 0, which is conflicted with *k*_0_ ≠ 0. Therefore, the assumption is wrong; that is, (11) is historical experience related.

**Definition 9 (group calibration function)**

Giving that *l*_*a*_ denotes the group where robot *R*_1_ belongs to, *l*_*b*_ denotes the group where robot *R*_2_ belongs to, *c* denotes the environment context, the group calibration function is expressed as

(13) $$\begin{array}{c} \tau \left(l_{a},l_{b},c\right)=\{\begin{array}{cc} \frac{m_{0}}{1+m_{1}\sqrt{\left(\Omega_{D_{1}}+\Omega_{D_{2}}\right)/2}} & l_{a}\neq l_{b}\\ 1 & l_{a}=l_{b}. \end{array} \end{array}$$

If *Ω*_*D*_1__ = NULL or *Ω*_*D*_2__ = NULL, then *τ*(*l*_*a*_, *l*_*b*_, *c*)∈[0.5–0.8]. 0 < *m*_0_ ≤ 1, 0 < *m*_1_ ≤ 1, *m*_0_ denotes the positive coefficient, *m*_1_ denotes the negative coefficient. The typical parameters of groups are given in Table 1.

*Characteristic 4* (newly joined robot reward). According to (13), if a robot does not belong to a group or has no collaboration with any robot in the group, the group reputation of the robot is NULL. When cooperating with the other robots, *τ*(*l*_*a*_, *l*_*b*_, *c*)∈[0.5–0.8], which stands for relatively high reputation.

## 4. Experiments and Results Analysis

Many simulation platforms can be used for multirobot systems [23], for example, Player/Stage, TeamBots, Gazebo, USARSim, Webots, Microsoft Robotics Studio, Swarmbot 3D, Swarmanoid Simulator, and so forth. In this paper, Player/Stage is selected as the simulation platforms because of the simple operability and flexibility. Player/Stage [24] was developed by Robotics Research Lab in University of Southern California in 1999, which is an open-source project that provides internal interface and simulation environment for multirobot system. The platform can be modified and expanded by the researchers worldwide according to their requirements.

### 4.1. Simulation and Performance Analysis

Three robots *R*_1_, *R*_2_, and *R*_3_ are put in area 1 and with the thrust capability of 2*N*, 3*N*, and 5*N*, respectively. Their initial reputation values are 0.5, 0.9, and 0.7. Three boxes *D*_1_, *D*_2_, and *D*_3_ are put in area 2, which need moving thrust of 2*N*, 6*N*, and 4*N*, respectively. The moving condition is that the thrust provided by the robot is greater than the needed thrust of the box.

Robot *R*_2_ is given the highest reputation value who is responsible for sending arranged commands to *R*_1_ and *R*_3_. Assuming that assignment is given to one robot to push the box, the robot moves from area 1 to area 2, and then finds the homologous box and try to push it.

**Figure 3:**
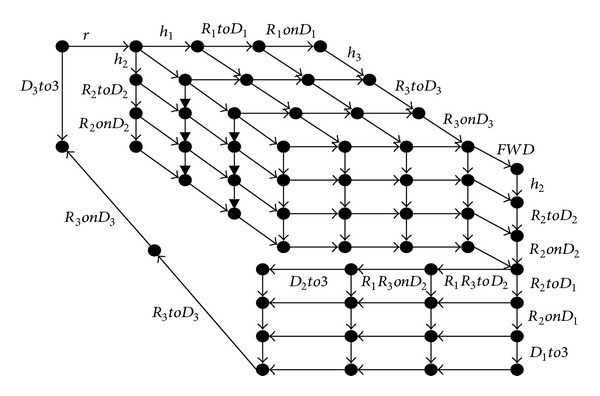
Task automata.

However, if *D*_2_ is too heavy to be pushed and needs cooperation from other robots, *R*_1_ and *R*_3_ are required to cooperate. The robot *R*_2_ goes to push *D*_1_, and after that *R*_1_ and *R*_2_ will go back to area 1. *R*_3_ returns to area 2, and continues pushing *D*_3_ to area 3. All of them return to the initial place waiting for next assignment. The process can be translated into task automata *A*_*s*_, shown in Figure 3.

The assigned task is to push the boxes to area 3. The local event set of each robot is presented as follows:

(14) E1={h1,R1  to  D1,R1  on⁡  D1,FWD,R1R3  to  D2,  R1R3on⁡D2,D2  to  3,r},E2={h2,R2  to  D2,R2  on⁡  D2,FWD,R2  to  D1,  R2  on⁡  D1,D1  to  3,r},E3={h3,R3  to  D3,R3  on⁡  D3,FWB,R1R3  to  D2,  R1R3  on⁡  D2,D2  to  3,  R3  to  D3,R3  on⁡  D3,D3  to  3,r}.

Checking that the decomposition conditions *E*_1_, *E*_2_, and *E*_3_, if *P*_1_(*A*_*S*_)||*P*_2_(*A*_*S*_)||*P*_3_(*A*_*S*_)≅*A*_*S*_, which certify that the task is decomposable and decomposed into three subtasks *P*_1_(*A*_*S*_), *P*_2_(*A*_*S*_), and *P*_3_(*A*_*S*_).

DTM presents the direct reputation relationships among robots. Three robots in the collaboration system named from *R*_1_ to *R*_3_, the DTM is shown as

(15) $$\begin{array}{c} \left[\begin{array}{ccc} 1 & 0.7 & 0.4\\ 0.7 & 1 & 0.8\\ 0.4 & 0.8 & 1 \end{array}\right]. \end{array}$$

**Figure 4:**
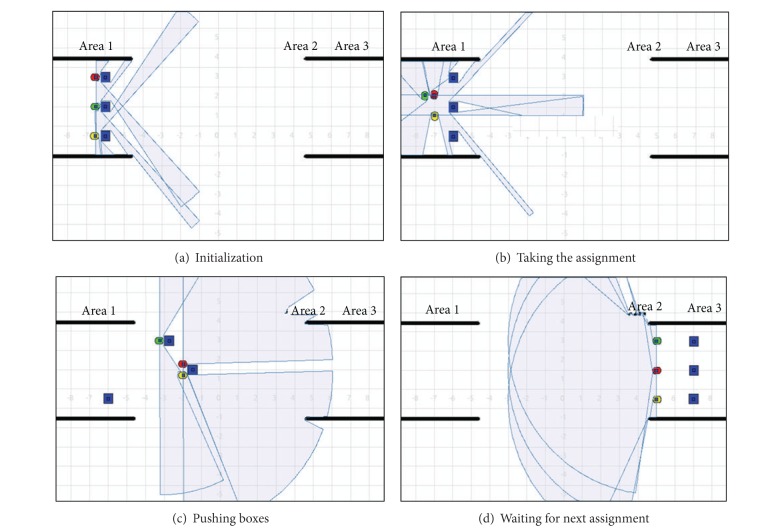
Simulation process of the approach.

The reputation in the matrix is updated after each step of the collaboration. Box moving is simulated by the approach, shown in Figure 4.

Two typical allocation algorithms are used to make contrast and evaluate the performance of the proposed method, which are sequence allocation and auction allocation. The simulation has seven robots in area 1 and 14 boxes in area 2, without obstacles between them.

**Figure 5:**
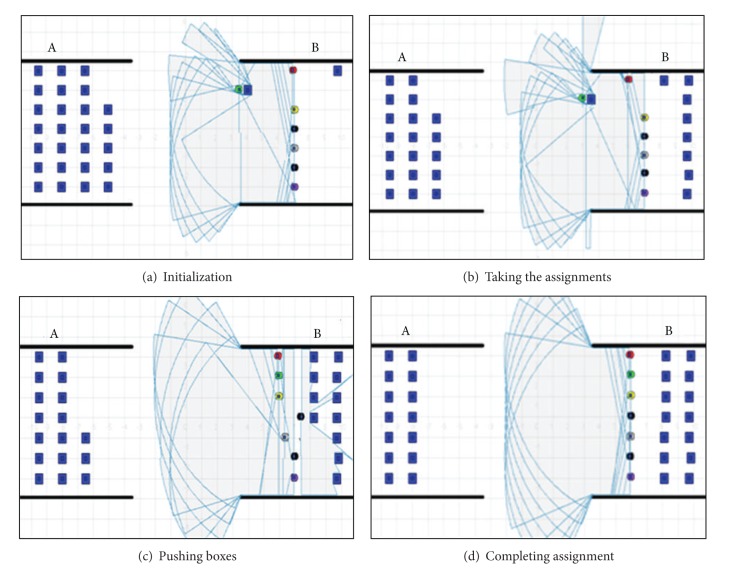
The simulation of sequence allocation.

In the case of the sequence allocations, the results of *N* mod 3 may be 1, 2, 3, 4, 5, 6, and 0. Tasks are assigned to the robot *R*_1_, *R*_2_,…, *R*_7_, respectively. The simulation process of sequence allocation is in Figure 5.

In the case of the auction allocation, the bidding is lauched at the beginning by the auctioneer. The best bid is picked out by the predetermined standard. The task will be assigned to the winner of the auction. The winner of the auction is chosen by the auctioneer giving the highest bid.

The bid matrix is used to store the value of robot biding for each assignment. A typical bid matrix in the simulation is presented as

(16) 0102030405060708091011121314R1571357109571357109R21281189131012811891310R310612101011151061210101115R411510111210141151011121014R513109121110131310912111013R6101481710912101481710912R7977209811977209811

**Figure 6:**
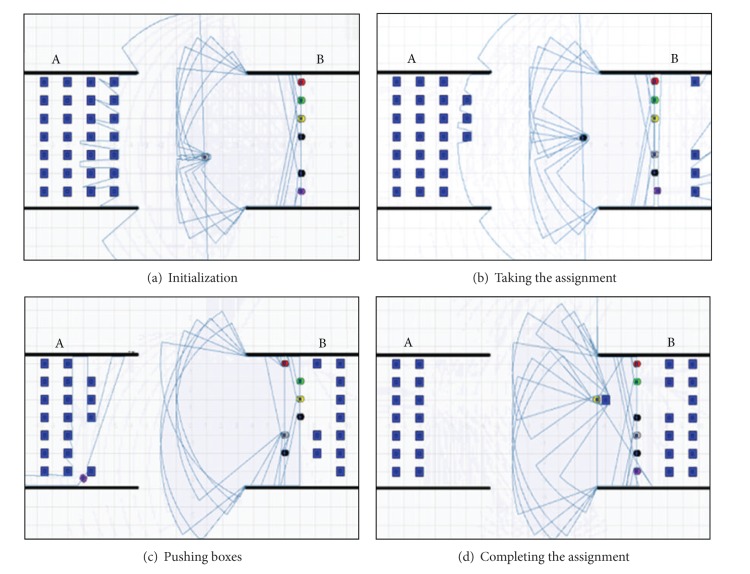
Simulation of auction allocation.

The tasks are performed in the following sequence: *R*_5_, *R*_6_, *R*_1_, *R*_7_, *R*_4_, *R*_2_, *R*_3_, *R*_5_, *R*_6_, *R*_1_, *R*_7_, *R*_4_, *R*_2_, *R*_3_. The simulation process can be described in Figure 6.

In the case of the task allocation by reputation, the initial reputation value of the system is the following matrix:

(17) [R1R2R3R4R5R6R7]T  =[0.810.610.550.480.830.460.72]T.

The DRM of seven robots is shown as

(18) R1R2R3R4R5R6R7R11.000.650.580.860.750.850.87R20.651.000.840.850.770.690.63R30.580.841.000.590.820.790.74R40.860.850.591.000.550.610.85R50.750.770.820.551.000.780.71R60.850.690.790.610.781.000.88R70.870.630.740.850.710.881.00

**Figure 7:**
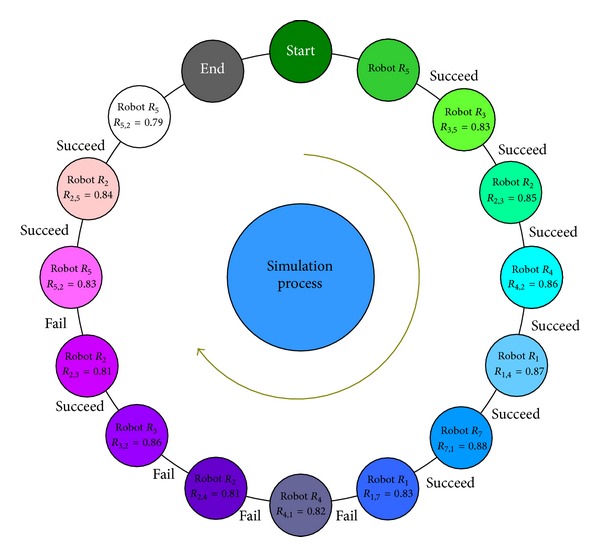
Process of task allocation by reputation.

The value of DRM is updated after each process of collaboration. If successfully finished the assigned task, the value of direct reputation between the robots increases by 0.01, otherwise reduces 0.05. In the simulation process, if the assigned task failed, the moving trajectory of the robot is the same to facilitate the performance comparison. The process of performing tasks is shown in Figure 7.

Every experiment is simulated six times to get a reasonable performance assessment. The ultimate results are the arithmetic average of the six times.

**Figure 8:**
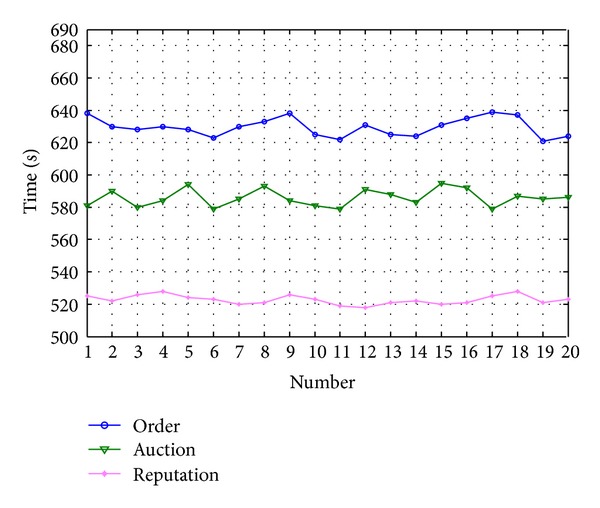
Comparison of the three allocation method.

The order allocation needs about 635.71 s to complete the assigned tasks. The auction allocation needs about 591.63 s to complete the tasks. The reputation-based allocation method needs about 525.15 s. The efficiency of task allocation by reputation is higher than the average of the other two methods, as shown in Figure 8.

### 4.2. Experimental Apparatus and Evaluation

**Figure 9:**
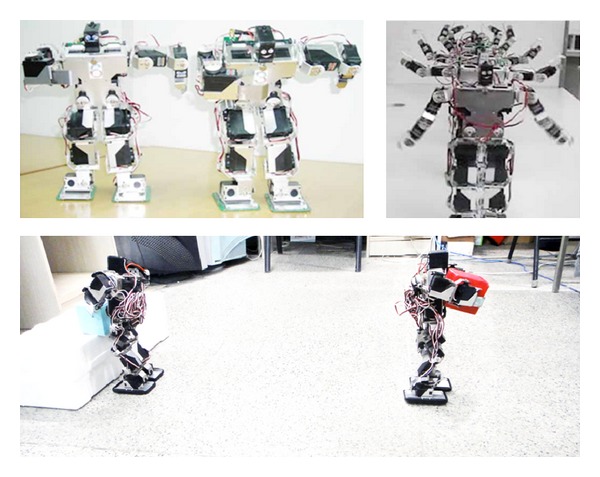
Biped-robot experimental apparatus.

The biped-robot apparatus are utilized as the experimental platform. The shape parameters of the robot are with upright height 33.3 cm, width 9.9 cm, arm length 15.9 cm, arms stretched flat horizontal length 41.7 cm, upper high 13.9 cm, waist high 19.4 cm, and weight 1 kg, as shown in Figure 9.

The wireless sensor module is used to send and receive commands, which are coded as the standard serial data and sent to the assigned robot. The effective transmission distance is about 30 m. The experimental parameters of task allocation are shown in Table 2.

Four task allocation methods are engaged to evaluate the efficiency. In addition to the proposed method, the other three methods are random allocation, order distribution, and simultaneous allocation.

A nonnumbered box with the size 8 cm^3^ is arranged to be moved to the destination. The moving distance is set to be 0.6 m. After receiving the task assignment, the robot moves to the side of the box, pick it up, move it, and put it down at the destination. The processes of picking up and putting down are done manually; other actions are done automatically.

For the case of task allocation using order distribution algorithm, the results of *N* mod 3 may be 1, 2, and 0. Tasks are allocated to the robot *S*_1_, *S*_2_, and *S*_3_, respectively based on the results, and every task allocation time interval is 30 s. For the simultaneous allocation algorithm, the allocation time interval is 0 s.

For the case of task allocation using random distribution, the random number set [0,1) is used. When the generated random number *R* ∈ [0–1/3), the task will be assigned to robot *S*_1_; if *R* ∈ [1/3–2/3), the task will be assigned to robot *S*_2_; if *R* ∈ [2/3–1), the task will be assigned to robot *S*_3_.

**Figure 10:**
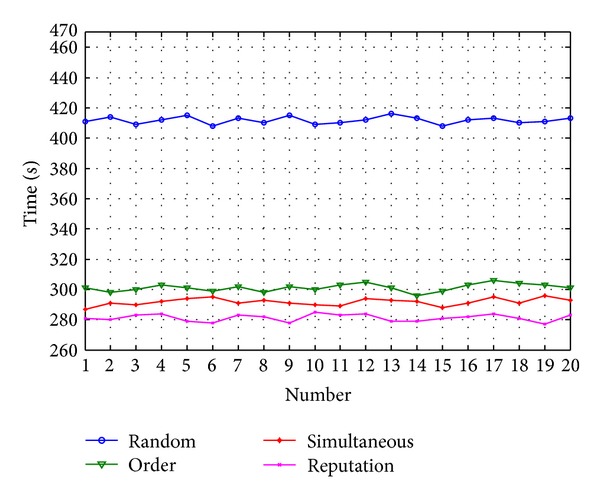
Comparison of small number of arranged tasks.

The tasks numbered *O*_1_, *O*_2_,…, *O*_*N*_ are generated by the task sequences. *N* stands for the number of tasks. *N* = 9 is taken, respectively, as the arranged tasks and each running time is 7 s. The arithmetic mean value of the maximum time and minimum time is removed to effectively prevent the unexpected result of random events. The results are shown in Figure 10.

**Figure 11:**
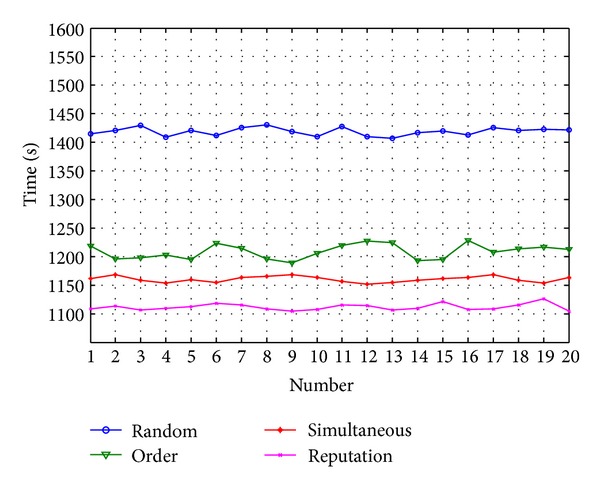
Comparison of medium number of tasks.

With the number of tasks increasing to *N* = 33, it is taken, respectively, as the arranged tasks (medium number tasks). The arithmetic mean value of the maximum time and minimum time are removed. The experimental results are shown in Figure 11.

According to the practical application, the robots with relatively high-speed capability are allocated more tasks and the efficiency of the task allocation by reputation is 4.35% higher than the second high method.

**Figure 12:**
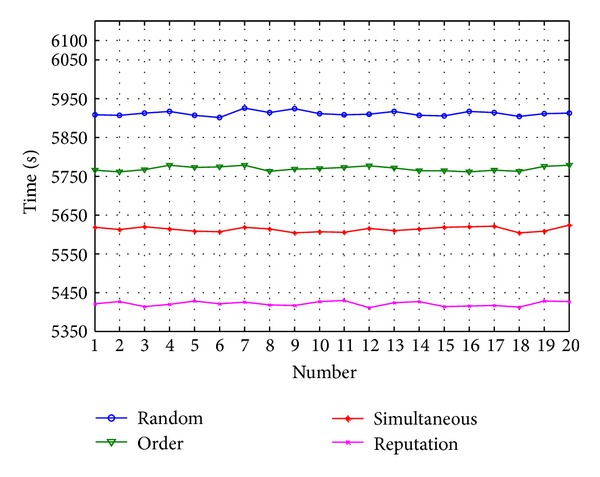
Comparison of large number of tasks.

When the number of tasks continues to increase to *N* = 180, which are taken, respectively, as the arranged tasks (large number of tasks). The test results are shown in Figure 12.

In the case of large number of tasks, the efficiency of task allocation by reputation is 3.57% higher than the second high method.

The experimental results show that the task allocation using reputation mechanism can effectively increase the performance and prevent a robot from a delay in the case of the individual robot failure.

## 5. Conclusion

Task planning is developed by two parts: task decomposition and task allocation. The processes of the task decomposition and task allocation using reputation mechanism, are presented. The robot collaboration strategy, the framework of reputation mechanism, and three reputations are defined in detail, which includes robot individual reputation, robot group reputation, and robot direct reputation. Time calibration function and group calibration function are designed to improve the effectiveness of the method, which are proved to be with characteristics of time attenuation, historical experience related, and newly joined robot reward. The success rate of collaboration is enhanced and the time of recovery and redistribution of the task are reduced.

Player/Stage is used as the simulation platform, and three biped-robots are established as the experimental apparatus. In the simulation, task decomposition is studied, and the result of task allocation is compared with the sequence and auction allocation methods. The biped-robots are used in the experiments, and four task allocation methods are engaged to evaluate the efficiency. The simulation and experimental results show that the approach can provide an effective performance for multirobot system.

## Figures and Tables

**Table 1:** Typical group parameters.

	Loyalty	Honesty	Improvement	Randomization
*m*_1_	0–0.1	0.1–0.2	0.2–0.3	0.4–0.6
*m*_0_	0.9–0.99	0.8–0.9	0.7–0.8	0.5–0.7

	Concealment	Tricky	Corruption	Deceivement

*m*_1_	0.6–0.7	0.7–0.8	0.8–0.9	0.9–0.99
*m*_0_	0.4–0.5	0.2–0.3	0.1–0.2	0–0.1

**Table 2:** The experimental parameters.

Parameters	Value
〈*κ*, *τ*, *ε*, Res〉	*κ*: four robots' identity Res: Transport, Movement
〈*R*, *M*, *S*, *T*, *P*〉	*R*, *M*: four robots *P*: logic set
[*r*_*b*_, *r*_*e*_]	*r*_*b*_: Time of box picked up *r*_*e*_: *r*_*b*_ + 30 s
own	0
*G *	*G*: four robots
*m*_0_, *m*_1_	0.6, 0.5
*α*, *β*, *θ*	1/3, 1/3, 1/3
